# Cavernous hemangioma of the mesorectum involving the rectum: a rare case report

**DOI:** 10.1186/s12876-021-01949-5

**Published:** 2021-10-15

**Authors:** Yan Zhang, Bing Wu

**Affiliations:** Department of Radiology, West China Hospital, Sichuan University, 37# Guoxue Road, Chengdu, 610041 China

**Keywords:** Hemangioma, Mesorectum, Radiology, Cavernous, Case report

## Abstract

**Background:**

Gastrointestinal hemangiomas are very rare and are even rarer in the mesorectum. It is not clear whether mesorectal hemangiomas originate in the bowel wall or in the mesorectum. For clinicians, to correctly identify the imaging features of mesorectal hemangiomas is important.

**Case presentation:**

We herein describe a case of a 31-year-old male that presented with hematochezia and sensation of rectal tenesmus. Both the rectal MRI and contrast-enhanced CT scan of the whole abdomen indicated rectal wall thickening, marked dilatation, and tortuous vessels around the rectum. In addition, a contrast-enhanced portal venous phase CT scan showed the dilation of portal vein, splenic vein and inferior mesenteric vein. The dilated inferior mesenteric vein extending down to the mesorectum, and became marked dilatation and tortuous vessels around the rectum. The patient underwent laparoscopic surgical resection of the mesorectal lesion and the involved portions of the rectum. The surgical samples underwent pathological analysis, and a diagnosis of cavernous hemangioma was confirmed. Seven days after surgery, the patient was discharged without postoperative complications.

**Conclusions:**

This case highlights the imaging features of mesorectal hemangiomas. In addition, in this current case, the mesorectal hemangioma more likely originated in the mesorectum.

## Background

Hemangiomas are benign proliferating vascular malformations, characterized by abnormal proliferation of blood vessels and mesenchymal tissue. They may be congenital or appear soon after birth [1]. Hemangiomas are often solitary lesions. The presence of multiple hemangiomas or those that extensively involve an organ or body part are termed hemangiomatosis [2]. Hemangiomas in the gastrointestinal system are very rare, accounting for only 0.05% of intestinal tumors [3, 4] and are even rarer in the mesorectum; this is the first reported case in the English literature to our knowledge.

Based on their pathology, hemangiomas are classified into three categories: capillary, cavernous, and mixed type, but the majority of hemangiomas in mesenteric regions reported thus far are of the cavernous type. No definite gender predominance has been identified. Patients can present at any age, but most present in young adults in the third decade of life [2].

It is not clear whether mesorectal hemangiomas originate in the bowel wall or in the mesorectum. Some reported cases of mesenteric hemangiomas have been thought to originate in the bowel wall and extend into the mesentery [3, 5–11]. In previous reports, hemangiomas have been mostly located in the small intestine mesentery [3, 5, 6, 8–11]. We herein report a case of mesorectum cavernous hemangioma involving the rectum with CT and MRI images.

## Case presentation

A 31-year-old male developed a sensation of rectal tenesmus one month before admission. He consulted the local hospital and proctitis was considered. The symptoms did not improve significantly after treatment. One week later, hematochezia suddenly occurred, resulting in blood loss of about 100 ml. The bleeding resolved without treatment. The patient was admitted to our hospital.

**Fig. 1:**
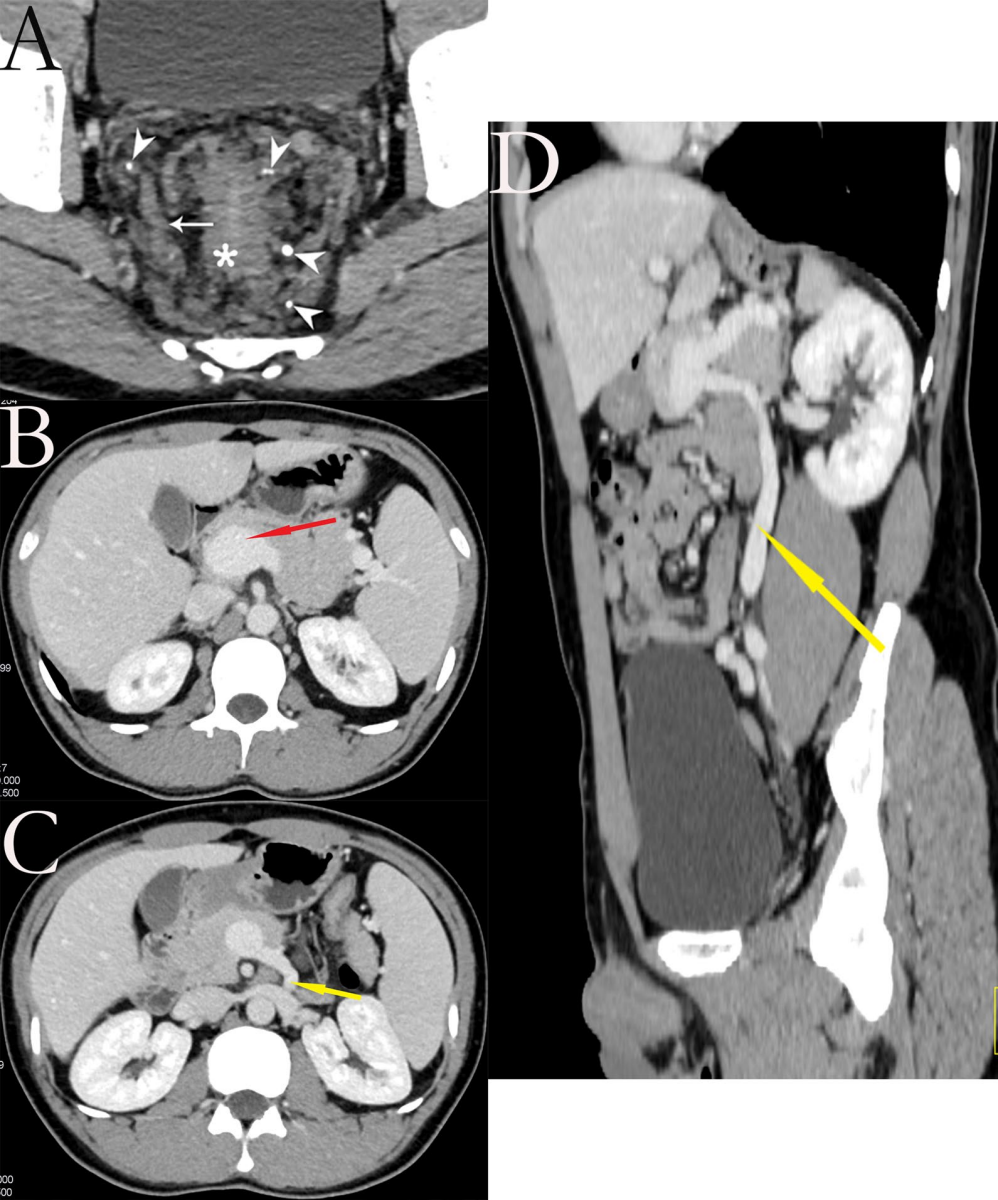
Contrast-enhanced portal venous phase CT scan. **A** Axial view: CT showing wall thickening of rectum (asterisk), dilatation and tortuous vessels around the rectum (arrow) with scattered calcifications (arrowhead). **B** Axial view: CT showing portal hypertension (red arrow). **C** axial view, **D** sagittal view: CT showing the dilatation inferior mesenteric vein (yellow arrow)

**Fig. 2:**
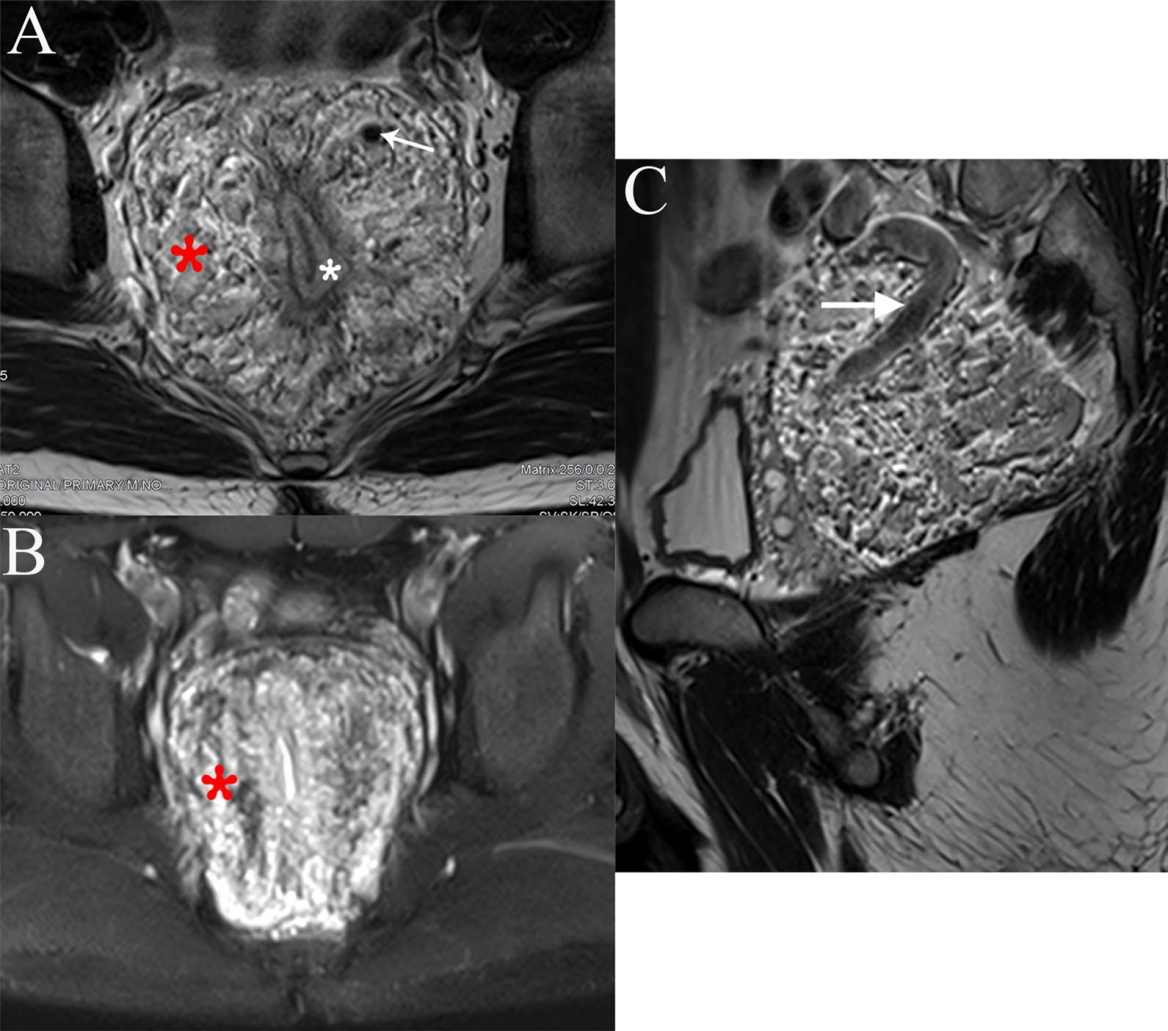
Rectal MRI. **A** Axial T2-weighted images showing wall thickening of the rectum (white asterisk), heterogeneous high signal intensity of the lesion (red asterisk) and black signal vessel (flowing void effect) (arrow). **B** Axial fat-suppressed T2-weighted images with the high signal intensity lesion more clearly depicted (red asterisk). **C** Sagittal T2-weighted images showing the dilatation inferior mesenteric vein (arrow)

His physical examination was unremarkable except for the digital rectal examination, which identified a soft mass in the right lateral wall of the rectum at 7 cm from the anal verge, about 3 cm × 3 cm in size, that grossly bled on exam. The complete blood count did not reveal anemia. A total colonoscopy revealed a discoid protuberance of mucosa at the rectum 7 cm from the anus, and erythema of the mucosal surface. The subsequent rectal biopsy indicated chronic inflammatory changes of the mucosa. A contrast-enhanced CT scan of the whole abdomen revealed rectal wall thickening, marked dilatation, and tortuous vessels around the rectum with scattered calcifications (Fig. 1A). In addition, a contrast-enhanced portal venous phase CT scan showed the dilation of portal vein and inferior mesenteric vein (Fig. 1B–D), and the dilated inferior mesenteric vein became marked dilatation and tortuous vessels around the rectum. The rectal MRI showed wall thickening of the involved portion of the rectum. A high signal intensity lesion was seen on T2-weighted sequences and was more clearly depicted with fat suppression (Fig. 2). Hemangioma of the mesorectum was considered after a multidisciplinary team discussion.

**Fig. 3:**
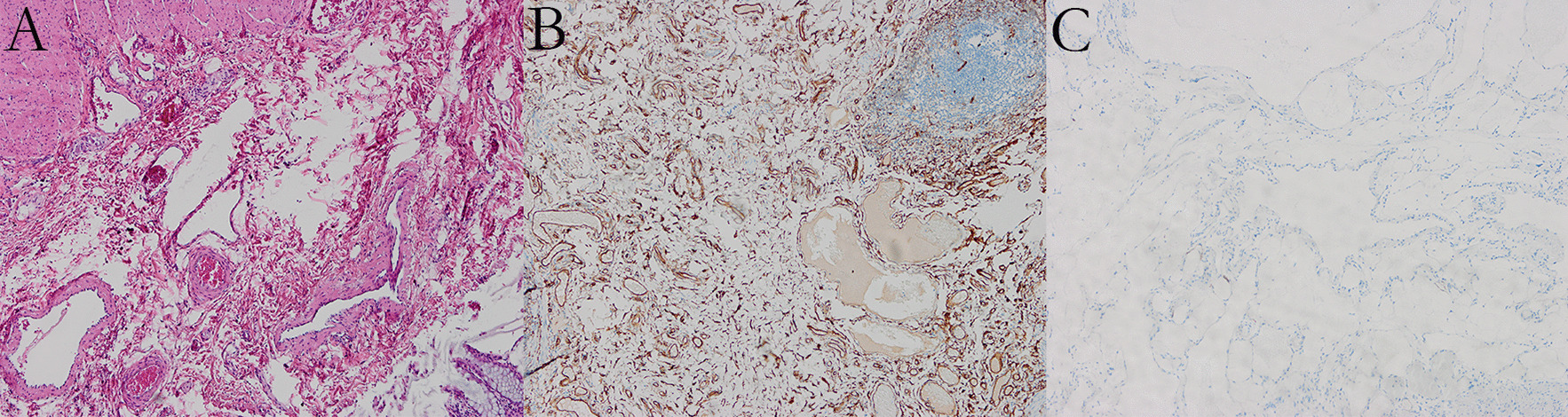
Histological and immunohistochemical pictures of the cavernous hemangioma. **A** H&E staining of the resected specimen revealing characteristic diluted blood vessels (× 100). The immunohistochemical results showing CD34 was positive in vascular endothelial (**B**), whereas D2-40 was negative (**C**) (× 100)

Three days after admission, the patient underwent laparoscopic surgical resection of the involved portions of the rectum and mesorectum, as well as a coloanal anastomosis. Surgical samples were taken and sent for pathological examination. The pathology viewings indicated a dark red area in the mesentery adjacent to the peritoneal reflection; the lesion was spongy and there was blood outflow from the incision. Histologically, the neoplasm was shown to be composed of dilated thin-walled blood-filled vascular spaces, and these spaces were lined by endothelial cells (Fig. 3A). The immunohistochemical results indicated CD34-positive results, whereas the lymphatic endothelial marker D2-40 was negative (Fig. 3B, C). The lesion was diagnosed as a cavernous hemangioma. The patient had no postoperative complications following laparoscopic surgical resection and returned home on postoperative day seven.

## Discussion and conclusions

Hemangiomas are a benign proliferative congenital vascular malformation that originate from the embryonic sequestrations of mesodermal tissue and can be found in any organ. They have an incidence of approximately 2–3% [2, 4]. While intestinal hemangiomas are well-known and have previously been described in the literature, mesorectal hemangiomas represent one of the rarest sites for hemangiomas. It is not clear whether mesorectal hemangiomas originate in the bowel wall or in the mesorectum. Some studies suggested that mesenteric hemangiomas may originate either from the bowel wall and the mesentery, or the mesentery alone [7]. We herein report a case of a mesorectal hemangioma that presented with rectal wall thickening, and the dilation of portal vein, splenic vein and inferior mesenteric vein. The dilated inferior mesenteric vein extending down to the mesorectum, and became marked dilatation and tortuous vessels around the rectum. These findings suggest that, in this case, the mesorectal hemangioma originated in the mesorectum and ultimately involved the rectal wall.

Clinical presentations and diagnoses of hemangiomas in mesenteric regions depend on their size, localization, and intestinal wall involvement. Smaller tumors are often asymptomatic whereas symptomatic masses tend to be large at the time of diagnosis. Larger hemangiomas located in mesenteric regions can cause intraperitoneal hemorrhage and can often result in life-threatening conditions. When hemangiomas involve the bowel wall, they may result in intraluminal bleeding. Therefore, the clinical manifestations are hematemesis or melena. Long-term chronic occult blood loss is an important cause of anemia [7, 12, 13].

Currently, radiological evaluation plays an important role in the noninvasive examination of abdominal diseases, including mesorectal hemangioma. Imaging examinations, such as CT and MRI, cannot be used to unequivocally diagnose hemangiomas in mesenteric regions, but they do allow a more precise evaluation of the size, vascularization, and possible involvement of adjacent structures of the tumor [7]. Calcifications were seen scattered throughout the lesion on CT. Some studies suggested that this was due to degenerative changes as a consequence of the thrombosis within the sinuses, caused by perivascular inflammation and stasis of blood flow [4, 10]. Other studies have shown that calcifications may ultimately form phleboliths, which represent an important diagnostic feature, seen in 26–50% of affected adult patients [4]. Phleboliths are usually signal voided on both Tl and T2 weighted images, and thrombosed vessels show serpiginous structures with high signal intensity on MRI [10]. An important imaging finding in our case suggested that a mesorectal hemangioma originated from the mesorectum rather than the bowel wall, which is different from some reported cases of mesenteric hemangiomas. A contrast-enhanced portal venous phase CT scan showed the dilation of portal vein, splenic vein and inferior mesenteric vein. The dilated inferior mesenteric vein extending down to the mesorectum, and became marked dilatation and tortuous vessels around the rectum. We guess this might be due to the increased blood flow in the cavernous hemangioma of the mesorectum, which leads to increased venous blood return. Image examination also revealed transmural thickening of the wall of the involved bowel loops. On CT hemangiomas may be homogeneous or heterogeneous with possible cystic structures. The heterogeneity of hemangiomas may be due to intralesional degeneration consisting of hemorrhage, fibrosis, or calcification [2]. On MRI, hemangiomas usually have an iso- or hypo-intense signal on T1-weighted images. On T2-weighted images, hemangiomas typically show high signal intensity and are more clearly depicted with fat suppression. Degenerative changes, such as fibrosis, result in a heterogeneous signal intensity on T2-weighted images. Intratumoral hemorrhage may show hypointense signal on T2-weighted images, making hemangiomas hard to differentiate from other solid mesenchymal tumors, such as fibromas or leiomyomas [4]. Characteristic discontinuous peripheral nodular progressive enhancement patterns can be seen in some mesenteric hemangiomas. However, enhancement may be heterogeneous or minimal or may be present only on delayed phases likely due to internal fibrotic changes [2].

A definitive diagnosis of cavernous hemangioma of the mesorectum depends on histopathological examination. Because of the high risk of hemorrhage, biopsy of clinically suspected hemangiomas is not recommended. Histologically, cavernous hemangiomas are composed of dilated vessels of varying sizes, which are lined with flattened endothelial cells with little fibrous connective tissue between the vascular channels. The vessel lumen often shows thrombosis and contains a large number of erythrocytes [9, 14, 15]. To avoid the risk of bleeding during biopsy, we used immunohistochemistry to diagnose our patient. The results were positive for CD34, but were negative for the lymphatic endothelial marker D2-40. In addition, CD31 is vascular endothelium-specific, and is often as a diagnostic marker of hemangioma [5, 9].

The optimal treatment of hemangiomas in mesenteric regions is surgical resection of the lesion and the involved intestinal segments [3, 8]. Recurrence after complete resection are rare. Other nonoperative techniques, such as low-dose radiation therapy, cryotherapy, brachytherapy, sclerotherapy or interventional angiography, often result in symptom recurrence and are therefore only temporary solutions [3, 4].

Mesorectal hemangiomas are extremely rare tumors, and it is not known whether they originate in the bowel wall or the mesorectum. Some studies have described mesenteric hemangiomas that originate in the bowel wall and extend into the mesentery. In this current case, the mesorectal hemangioma more likely originated in the mesorectum. We used CT imaging for diagnosis and to evaluate the size, vascularization and possible involvement of adjacent structures of the mesorectal hemangioma. We surgically resected the mesorectal lesions and affected intestinal segments. Recurrences after complete resection are rare, and we are confident that our patient will not experience a recurrence of this tumor.

## Data Availability

Not applicable.

## Abbreviations

MRI: Magnetic resonance imaging
CT: Computed tomography
